# Case report: ultrasound assisted catheter directed thrombolysis of an embolic partial occlusion of the superior mesenteric artery

**DOI:** 10.3389/fradi.2023.1167901

**Published:** 2023-05-18

**Authors:** Simone Bongiovanni, Marco Bozzolo, Simone Amabile, Enrico Peano, Alberto Balderi

**Affiliations:** ^1^ Interventional Radiology Unit, Department of Radiology, A.O. S. Croce e Carle - Cuneo, Cuneo, Italy; ^2^ Postgraduate School in Radiology, University of Turin, Turin, Italy

**Keywords:** ultrasound assisted catheter directed thrombolysis, superior mesenteric artery, EKOS, acute mesenteric ischemia, embolism, thrombolytic therapy, tissue plasminogen activator

## Abstract

Acute mesenteric ischemia (AMI) is a severe medical condition defined by insufficient vascular supply to the small bowel through mesenteric vessels, resulting in necrosis and eventual gangrene of bowel walls. We present the case of a 64-year-old man with recrudescence of prolonged epigastric pain at rest of few hours duration, cold sweating and episodes of vomiting. A computed tomography scan of his abdomen revealed multiple filling defects in the mid-distal part of the superior mesenteric artery (SMA) and the proximal part of jejunal branches, associated with small intestine walls thickening, suggesting SMA thromboembolism and initial intestinal ischemia. Considering the absence of signs of peritonitis at the abdominal examination and the presence of multiple arterial emboli was decided to perform an endovascular treatment with ultrasound assisted catheter-directed thrombolysis with EkoSonic Endovascular System—EKOS, which resulted in complete dissolution of the multiple emboli and improved blood flow into the intestine wall. The day after the procedure the patient's pain improved significantly and 5 days after he was discharged home asymptomatic on warfarin anticoagulation. After 1 year of follow-up the patient is fine with no further episodes of mesenteric ischemia or other embolisms.

## 1. Introduction

Acute mesenteric ischemia (AMI) is a severe medical condition defined by insufficient vascular supply to the small bowel through mesenteric vessels, resulting in necrosis and eventual gangrene of bowel walls: for these reasons is a potentially life-threatening condition with comparable urgency to myocardial infarction or stroke.

Heart failure, atrial fibrillation, coronary heart disease (CHD), arterial hypertension, and peripheral arterial disease (PAD) are predisposing risk factors (1). Etiology of mesenteric ischemia include mesenteric arterial occlusion caused by thromboembolism (50%), atherosclerotic disease (15%–25%), mesenteric venous thrombosis (5%–15%) and vasospasm or global hypoperfusion including hypotension and shock (2).

The landmark symptom for the vast majority of patients is abdominal pain which is usually sudden in patients with AMI caused by emboli, and instead has insidious onset in patients with thrombotic etiology where the atypical clinical presentation of pain often postpones the diagnosis, worsening the prognosis (3). Mortality rate is about 30%–70% and the dominant factor is delay in diagnosis despite vast clinical experience and recognition of this entity, if treated during its initial stage its mortality rate is less than 30% (4).

Treatment options depends on the etiology, hemodynamic stability of the patient and the experience and expertise of the treating team (5). Surgical embolectomy is considered the gold standard; however, innovative diagnostic and endovascular procedures have been proposed in recent years as possible therapeutic tool. Early endovascular treatment has the potential advantage of in some cases precluding laparotomy or reducing the extension of enterectomy, as it could theoretically restore intestinal perfusion in less time than open thrombectomy, patch arterioplasty or surgical bypass (4).

## 2. Case description

A 64-year-old man, with a history of transient ischemic attack and hypertrophic cardiomyopathy with an implantable cardioverter-defibrillator (ICD) and in therapy with atenolol 50 mg/day, presented to our Emergency Department for recrudescence of prolonged epigastric pain at rest of few hours duration, cold sweating and episodes of vomiting. The reported pain has been present for about 1 year under effort (walking, climbing stairs) with remission at rest. The patient wasn't on any anticoagulation therapy at the time of admission. The patient was hemodynamically stable with a heart rate of 118 bpm controlled by the pacemaker and a blood pressure of 160/80 mmHg. Cardiologic evaluation revealed symmetrical pulses, no murmurs at the auscultation and no abnormal findings were found on echocardiography. An abdominal examination revealed minimal epigastrium tenderness with no signs of peritonitis. A laboratory examination revealed a white blood cell (WBC) count of 12.3 K/μl, a serum C-reactive protein of 4.2 mg/L, a serum lactate on venous blood gas of 3.1 mmol/L, a serum troponin I of 1,143.4 pg/ml and on second control a serum troponin I of 1,117.2 pg/ml.

The patient was hospitalized in coronary care unit where he showed poor response to gastroprotective and antacid drugs. The movement of troponin curve wasn't compatible with a pattern of myocardial infarction but referable to chronic myocardial damage related to the known hypertrophic cardiomyopathy.

**Figure 1:**
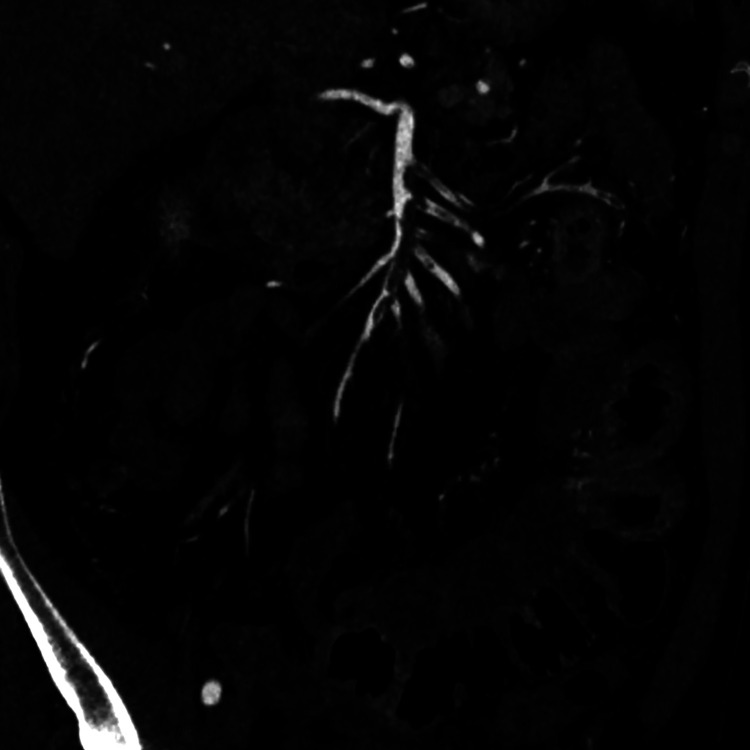
Pre-treatment arterial phase CTA—maximum intensity projection reconstruction showing multiple filling defects in the mid-distal part of the superior mesenteric artery (SMA) and in the proximal part of jejunal branches.

Due to the persistence of symptomatology 36 h after the admission in emergency room a contrast-enhanced computed tomography scan of the abdomen was performed, and the arterial phase revealed multiple filling defects in the mid-distal part of the superior mesenteric artery (SMA) and in the proximal part of jejunal branches with no evidence of atherosclerosis, associated in portal phase with small intestine walls thickening without intramural or extramural air, suggestive for SMA thromboembolism and initial intestinal ischemia (Figure 1). Additional laboratory tests were done which showed an increase of WBC count 18.7 K/μl, a serum troponin I of 1,244.6 pg/ml and a serum lactate on venous blood gas of 0.9 mmol/L.

A multidisciplinary evaluation with general surgeon, vascular surgeon and interventional radiologist was done and considering the absence of signs of peritonitis at the abdominal examination, the patient was sent to the angio suite. The option of open surgery was also considered but the lack of physical findings and the presence of multiple arterial emboli prompted us to attempt endovascular treatment. In case of any clinical deterioration the patient would have undergone an open intervention.

**Figure 2:**
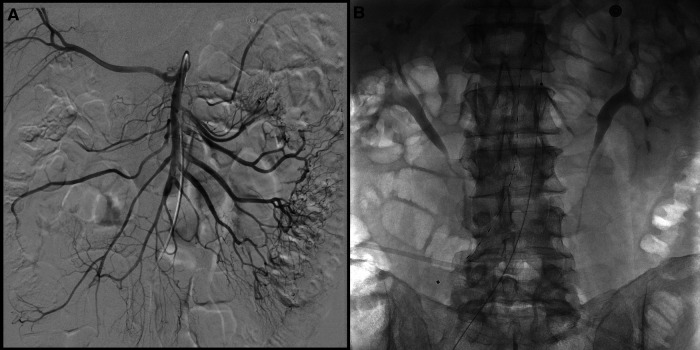
(**A**) Pre-treatment DSA images showing multiple filling defects in the mid-distal part of the superior mesenteric artery (SMA) and obstruction of at least two jejunal branches with still a perfusion of intestine loops by collateralization. (**B**) EKOS catheter 18 cm positioned in SMA.

The patient underwent ultrasound-guided right femoral artery puncture after local groin anesthesia with 10 ml of lidocaine and a 6-Fr introducer sheath was inserted over a J-wire. Subsequently, a Cobra-C2 catheter (Cook Medical, Bloomington, IN, USA) was inserted into the superior mesenteric artery. An angiographic study was done which showed the presence of subtotal thrombotic occlusion of the SMA with multiple emboli in jejunal branches and still a hemodynamic compensation by collateralization (Figure 2). Due to these characteristics of the embolic material was decided not to proceed with mechanical thrombectomy because of the high risk of iatrogenic distal embolism and the difficulty to catch all the emboli in distal branches. Therefore, it has been decided for endovascular revascularization using EkoSonic Endovascular System—EKOS (Boston Scientific, Marlborough, MA, USA). Through the Cobra-C2 catheter a 0.035-inch Glidewire Advantage® guidewire 260 cm length (Terumo Corporation, Tokyo, Japan) was inserted into the SMA and using an exchange maneuver the EKOS catheter with a 18 cm treatment zone was placed into the SMA. The catheter was connected to the ultrasound generator and to the infusion pump of recombinant tissue plasminogen activator (rtPA) at a rate of 1 mg/h for the thrombolysis. A concomitant systemic anticoagulation therapy with heparin was done with a target of activated Partial Thromboplastin Time (aPTT) ratio of 1.8. The treatment continued for 12 h with the patient's hemodynamic stability.

**Figure 3:**
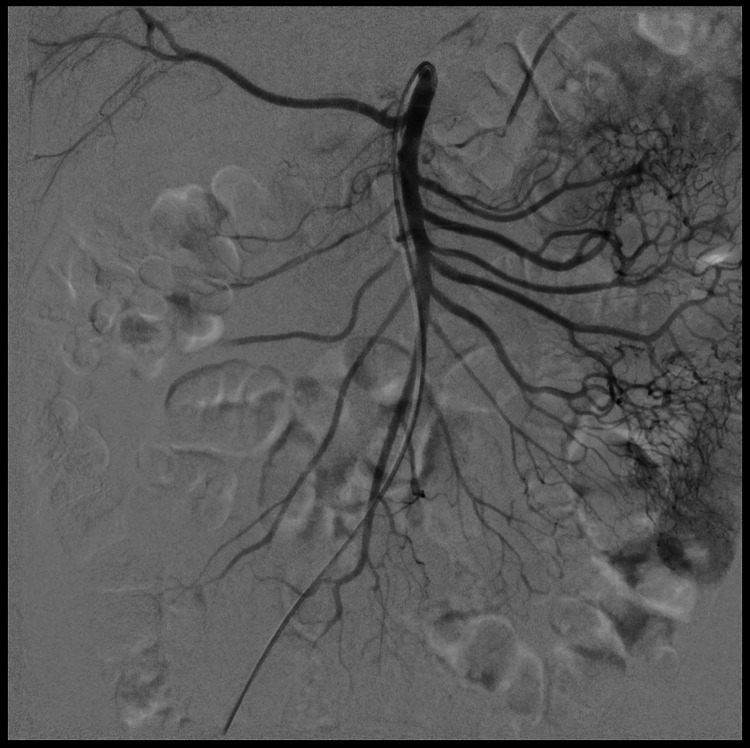
Post-treatment DSA images showing complete dissolution of the emboli with an improvement of the blood flow into the intestine wall.

After the therapeutic treatment period the patient's pain improved significantly and the EKOS catheter was removed. The patient returned into the angio suite the following morning to repeat an angiogram. The SMA angiographic study confirmed the absence of vessel stenosis and revealed a complete dissolution of the multiple emboli with an improvement of the blood flow into the intestine wall (Figure 3).

**Figure 4:**
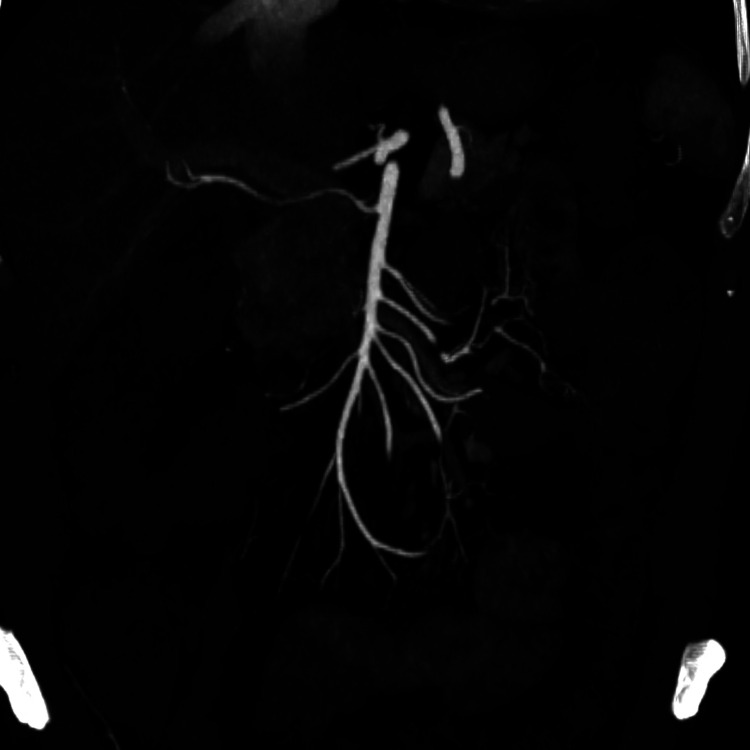
Post-treatment arterial phase CTA—maximum intensity projection reconstruction showing superior mesenteric artery without any filling defects of thrombotic nature.

Four days after the procedure the patient underwent an abdominal CT scan that confirmed the previous angiogram images without any filling defects of thrombotic nature of the upper mesenteric artery, thoracic aorta, and large splanchnic vessels (Figure 4). Compared to the CT exam before the treatment, there was also an important reduction in the parietal thickening of the multiple intestinal loops.

The patient was discharged home the day after asymptomatic on warfarin anticoagulation, beta-blockers, and large spectrum antibiotic therapy.

After 1 year of follow-up the patient is fine with no further episodes of mesenteric ischemia or other embolisms.

## 3. Discussion

The optimal management of AMI remains controversial, while many authors suggest the surgical approach with concurrent assessment of bowel viability as the first line treatment, others consider endovascular approach a superior alternative. Usually, the choice of the first therapeutic option appeared mostly dependent on surgeon or interventional radiologist expertise and institutional preference, confirming lack of a standardized protocol (6). Furthermore, the treatment options depend on the etiology, the presence or absence of peritonitis and on hemodynamic stability of the patient.

The surgical treatment consists in exploratory laparotomy with the assessment of bowel viability, removal of the non-viable bowel tract and in case of embolism in SMA the restoration of blood flow by mechanical embolectomy with a Fogarty catheter. The endovascular treatment consists in a mechanical embolectomy by thromboaspiration or stent-retriever or by the combination of these two techniques. While surgery is required in cases of non-viable bowel, minimally invasive interventional approaches have several advantages, such as the avoidance of general anesthesia and the use of laparotomy in high-risk patients. However, the disadvantage is that some useful time could be lost, a delayed surgical intervention place additional bowel at risk due to only partial resolution of the obstruction to blood flow (7).

In this reported case the patient had a recent onset of abdominal pain with no peritoneal signs and a suspected cardiac embolism origin. The abdomen CT scan showed wall thickening and hyperemia in multiple small bowel loops without intramural or extramural air, the SMA presented multiple filling defects in the mid part and in proximal side branches with no evidence of atherosclerosis. According to the recently published update of the ACR criteria of the radiologic management of mesenteric ischemia, in our case is considered appropriate an endovascular approach. The ACR criteria also suggest that a transcatheter thrombolysis may be considered if aspiration embolectomy fails (8).

In this case, in presence of a partial occlusion of the SMA and the presence of multiple distal emboli with still a blood flow to the intestinal loops, has been decided to proceed directly with catheter thrombolysis using EKOS.

The EkoSonic Endovascular System is designed for ultrasound assisted catheter-directed thrombolysis. The 0.035-inch guidewire compatible catheter allows selective and controlled delivery through its multiside hole ports directly to the affected vascular segments of high concentration of thrombolytic agent (Alteplase 1 mg/h), high frequency ultrasound increase the permeability of the thrombolytic agent into the thrombus disassociating the fibrin strands, increasing thrombus surface area and making more plasminogen activator receptor sites available for facilitating thrombolysis (9, 10). This device is FDA approved for selective and controlled infusion of thrombolytic agents into occluded peripheral vessels, including pulmonary embolism, deep vein thrombosis, and peripheral artery occlusions. In massive acute pulmonary embolism, EKOS has shown a higher complete thrombus resolution rate (100% vs. 50%), reduced thrombolytic treatment duration (17.4 vs. 26.7 h) and hemorrhagic complications (0% vs. 3%) due to the lower dose of thrombolytic agent administered compared with catheter-directed thrombolysis alone (11). The EKOS system can be used for a maximum treatment time of 24 h. In acute pulmonary embolism, as evidenced by SEATTLE II and OPTALYSE PE trial, improved right ventricular function and reduced clot burden were obtained after 6–12 h of treatment (10, 12). In our case was decided to treat the patient for 12 h but there is no clear data about the correct treatment time with this device in acute mesenteric ischemia. Indeed, only two case report were described in literature about ultrasound-assisted catheter directed thrombolysis but none with this patient characteristics (7, 13).

In conclusion this case illustrates the successful applications of ultrasound assisted thrombolysis to dissolve a SMA embolic thrombus in patient with acute mesenteric ischemia. While, this procedure can't be used to perform a rapid restoration of the intestinal blood flow, in some specific case with a partial occlusion of the SMA and a residual blood flow to the intestine loops can be employed successfully. Thus, the limitations of this procedure are prolonged treatment duration, risk of bleeding and need to return to the angio suite for the evaluation of the therapy. The advantages include being a minimally invasive procedure without the need of a general anesthesia of a long operation in these high-risk patients.

This device can represent a new therapeutic option also in these characteristic cases of acute mesenteric ischemia with widespread thrombosis but presence of residual flow and no signs of peritonitis. Larger case series are needed to confirm this result, to establish proper patient selection and treatment timing.

## Data Availability

The raw data supporting the conclusions of this article will be made available by the authors, without undue reservation.
